# 

**Published:** 1888-04-14

**Authors:** 


### Special Notice to Newsagents, Advertisers, and the Trade.

### CHANGE OF OFFICE AND PUBLISHER.

Notice is hereby given that the Office of The Hospital will henceforth be at 38, Old Jewry (Cheapside end), E.C., to which address all communications should be sent. Mr. A. Doig has been appointed Advertising Agent, and Mr. J. H. S. Hanning, Manager. All Cheques and Post Office Orders should be made payable to J. H. S. Hanning, 38, Old Jewry, E.C. crossed Lloyds, Barnetts, and Bosanquets Bank (Limited).

### To Secretaries and Matrons.

The Editor will be glad to have a copy of the Regulations, and also of every Report issued from the Press of Asylums, Hospitals, Convalescent and Nursing Institutions, as well as those of other Charities, so soon as they are published, and an early copy in duplicate of all appeals for funds. Notices of Annual and other Meetings, Festival Dinners, etc., will be welcomed. All such communications should be addressed to *The Editor, The Lodge, Porchester Square, London, W.* Any superintendent, secretary, matron, or other chief official who will regularly send such documents and information will be registered to receive free of cost a cover for binding The Hospital in half-yearly volumes on sending name and address to the publisher.

### Amusements with Profit for all Ages.

**Rules.**—All answers must be addressed to the Prize Editor, The Hospital, 38, Old Jewry, Cheapside, London, E.C., not later than the *first* post on Thursday next. The full name and address must in all cases be given, but a *nom de plume* may be added for publication. Only one side of the paper must be written on.

### FOR MEN AND WOMEN.

**Rule.**—Competitors can enter for *all* quarterly competitions, but no competitor may take more than *two quarterly prizes* during the year.

### Acrostic Competition.

Second quarter commenced February 25th, 1888; ends May 19th, 1888.

A PRIZE of ONE GUINEA will be given to the competitor who gains the highest number of marks for best original acrostics during the ensuing quarter. All acrostics sent in for this competition will be credited according to merit, twelve marks being the highest score given for one acrostic.

A PRIZE of HALF-A-GUINEA to be given to the competitor who gains the highest score for solution of acrostics set. *One mark* only will be given to the competitors who solve all the lights of each acrostic.

Exception will be made in the case of *quotation acrostics*, when *two marks* will be given, *one* for the correct solution of all *lights*, and *one* mark for the *source* of the quotations.

This week credit will be given for

**No. 4 Original Double Acrostic.**

### Results of No. 3 Original Double Acrostic.

Brownie (12), A. M. E. (11), Patience (11), Mater (8).

Erratum.—By a misprint "Byron" was given as the source of Light 2 in Double Acrostic No. III. "Pope" is the author of the quotation.

### (A) THE WORD COMPETITION.

Second Quarterly Competition commenced
February 4th, 1888; ends April 28th, 1888.

Three prizes of one guinea, 15*s.* and 10*s.* 6*d.* will be given each quarter to the three competitors who obtain the highest number of marks; (1) by making the largest number of words derived from the word or phrase set for anatomical dissection; or (2) for the best answers to (B); or (3) by (1) and (2) combined.

We shall give the newspapers and periodicals for dissection during this quarter, that for this, the *eleventh* week of the quarter, being

"Whitehall."

Observe.—Proper names, abbreviations, foreign words, words of less than four letters, and repetitions are barred; plurals, and past and present participles, of verbs are allowed. Nuttall's *Standard* dictionary only to be used.

N.B.—All words sent in must be arranged *alphabetically*, with correct total affixed.

### Results of Ninth Week.

*Cornhill.*—Patience (30), Dunce (30), Gretta (30), Sym (29), E. E. (29), Lightowlers (27), Lauriston (27), Butterfly (26), Ellice (26), Brisbane (26), Lodge (26), Oliver (22), Nelle (21).

### (B) THE LITERARY COMPETITION.

Some people hate puzzles of all kinds, including word competitions, but they are fond of correspondence, and many sisters and mothers are continually receiving and sending letters to and from members of the family who are abroad in India or the Colonies. The constant coming and going between England and other portions of the British Empire make it a matter of interest and importance to know something about the life, the climate, the amusements, the adventures, the clothing, and the surroundings of those who seek a livelihood beyond the seas. We shall therefore try to find space for the publication of selected letters from abroad, which may be sent by any of our readers who have friends and relations in foreign lands. We shall give marks for these letters as well as for the word competitions which will count equally for these prizes, so that anyone may enter for both or either, the prizes to be given to those who get the highest number of marks.

We have received this week letters for in sertion in this column from *Patience*, *Gretta*, and *Oliver*, whose letter we insert below.

### Letters from Across the Sea.

CALCUTTA.

We went, the other day, on quite a novel expedition. It was to see some schools in two or three native villages about thirty miles from Calcutta. We had to drive the whole way in "ticca gharries" (hackney carriages), and our bedding and provisions followed in a buffalo cart. The road was very good for about seventeen miles, and after that it was simply a track on the top of the embankment that divided the rice-fields. A tremendous storm of thunder and rain came on while we were still on our way; the road became a Slough of Despond, and we went at the rate of two miles an hour. We reached our destination—the inspector's bungalow—at eleven o'clock at night, and found both rooms occupied by native engineers. They obligingly vacated one room, and then to our horror we discovered that the buffalo-cart had not arrived. We had to make the best of it by sleeping two in the bed and two on the table. Fortunately, we had brought some provisions with us, so the next morning we were not starved for want of breakfast. We set off in palanquins to visit the schools, and before we had gone far we came upon the buffalo-cart. The servants had a dismal tale to tell of all their misfortunes: how the buffaloes had tried to get into the ditches, which were filled with water during the storm; how they had all got wet through in the rain; how the buffaloes at last became unmanageable, and rushed headlong into the water, cart and all; how finally the buffaloes were unyoked, the cart hauled out, and the men spent a miserable night by the roadside. We found afterwards that this was not all literally true, for they had left the cart and buffaloes in the road, and had passed the night very comfortably at a neighbouring village. We journeyed quite seven miles in our palanquins across rice-fields before we came to the village in which were the schools we wished to see. We have always found that these small village schools flourish better when English ladies take an interest in them, and this was the reason of our visit. We went to the girls' school and to the boys' school, and at the latter the master made a speech in English, a speech that had evidently been carefully studied and rehearsed for the occasion. Every sentence began with "Blessèd be the day," and was something in this style: "Blessèd be the day when these English ladies have come into our midst. Blessèd be the day when these daughters of the race of our conquerors have condescended to visit us." The secretary of the school then requested us "to put our shoes under his humble roof," by which he meant that he wanted us to enter his house and have something to eat. We found a regular native dinner prepared for us, but our hosts would not come into the room where we were eating, for it would have been pollution to them; they waited in the verandah, and took care that we had everything we wanted. We went back in our palanquins to the bungalow in the evening, and the next morning we sallied out again. Our experiences were very much the same as on the previous day. We returned to Calcutta on the fourth day, and as the roads were dry again we did the journey in a much shorter time than we expected.

### THE CHILDREN'S CORNER.

PRIZES.
FOR MOST CORRECT ANSWERS TO PUZZLES.
This Commenced October 1st, 1887.
One Pound a Month.
A PRIZE OF THREE GUINEAS
to the Competitor who shall have sent in the largest number of correct or best answers during the twelve months.

### Puzzles—Second Week.

No. 5. Find the four poets transposed below, and add to each the name of one of their best known works:

1. YESLLHE.
2. SNOYNENT.
3. GNOFWLLOEL.
4. DROSWWHROT.

No. 6. Square Word.—1. A musical instrument. 2. A South American plant. 3. Help to the drowning. 4 A distinguished British statesman of the nineteenth century.

No. 7. Double Acrostic.—Initials and finals form the name of two celebrated battles fought in the eighteenth century. 1. A celebrated Norwegian the conqueror of Normandy. 2. Farewell. 3. The founder of Islam. 4. One of Sir Walter Scott's heroes. 5 An Archbishop of Canterbury who is said to have divided the Bible into chapters and verses. 6. The founder of the Jesuits. 7. A material indispensable to artists. 8. A country we all love. 9. Famous Druidical remains.

Charade: Vindictive fury is my *first*;
And solid earth my *second*;
My *whole*, by Britain never seen,
Is Britain's sister reckoned.

### Results of Fifth Week Puzzles.

No. 17. Historical Charade. Van-dals who conquered Africa under Genseric.

No. 18. Enigma. Liquor-ice = Liquorice.

No. 19. LIVE = Live.

No. 20. Geographican Acrostic.

I ceber G

C anut E

E l Y

L ollard S

A nn E

N amu R

D ougla S

*All right.*—Jumps, A. C. K., Gem, Squeaker, Scrooge, Bagshot. *Three right.*—Hercules, Plummy Fluff, Mount Edgecumbe, Spider.

### Results of Sixth Monthly Competition.

*Jumps* and *Squeaker* having gained the highest number of marks during the past month, we divide the prize of £1 equally between them.

*Jumps* (Master Thomas A. McIntyre, 87, Grosvenor Road, London, S.W.), 10*s.*

*Squeaker* (Master C. Arnott, The Rectory, Beckenham, Kent), 10*s.*

### Conditions.

I. Every paper sent in must be clearly written, and one side only must be used. All competitors must mark at the head of their papers the number of their competition.

II. Each paper must be signed by the author with his or her real name and address. A *nom de plume* may be added, if the writer does not desire to be referred to by us by his real name. In the case of all prize-winners, however, the real name and address will be published.

III. All communications relating to prize competitions should be addressed to "The Prize Editor, The Hospital, 38, Old Jewry, London, E.C." Competitors are requested not to include in letters, etc., to the Prize Editor communications on matters of other business. All letters must be fully prepaid.

IV. The Prize Editor of The Hospital is the sole judge of the competitions, and his decision is in all cases final and without appeal.

V. The Prize Editor reserves the right to publish any paper sent in, whether it receives a prize or not. He does not hold himself responsible for any MSS. sent, nor can he undertake to return rejected competitions.

VI.—All children resident at school are requested to forward their *home* address in addition to the school one, when entering for the "Children's Corner" Competitions.

**Notice to all Correspondents.**—N.B.—All letters referring to this page, which do not arrive at 38, Old Jewry, Cheapside, London, E.C., by the *first post on Thursdays*, and are not addressed PRIZE EDITOR, will in future be disqualified and disregarded.

No one will be permitted to win the Monthly Prizes twice in any one year, the Annual prizes being offered especially to prevent this.

